# A general framework for automatic closed-loop control of bladder voiding induced by intraspinal microstimulation in rats

**DOI:** 10.1038/s41598-021-82933-7

**Published:** 2021-02-09

**Authors:** Abolhasan Yousefpour, Abbas Erfanian

**Affiliations:** Department of Biomedical Engineering, School of Electrical Engineering, Iran Neural Technology Research Center, Iran University of Science and Technology (IUST), Tehran, Iran

**Keywords:** Biomedical engineering, Spinal cord, Bladder

## Abstract

Individuals with spinal cord injury or neurological disorders have problems in voiding function due to the dyssynergic contraction of the urethral sphincter. Here, we introduce a closed-loop control of intraspinal microstimulation (ISMS) for efficient bladder voiding. The strategy is based on asynchronous two-electrode ISMS with combined pulse-amplitude and pulse-frequency modulation without requiring rhizotomy, neurotomy, or high-frequency blocking. Intermittent stimulation is alternately applied to the two electrodes that are implanted in the S2 lateral ventral horn and S1 dorsal gray commissure, to excite the bladder motoneurons and to inhibit the urethral sphincter motoneurons. Asynchronous stimulation would lead to reduce the net electric field and to maximize the selective stimulation. The proposed closed-loop system attains a highly voiding efficiency of 77.2–100%, with an average of 91.28 ± 8.4%. This work represents a promising approach to the development of a natural and robust motor neuroprosthesis device for restoring bladder functions.

## Introduction

Bladder hyper-reflexia or detrusor underactivity and dyssynergic contraction of the urethral sphincter are common clinical conditions affecting individuals with spinal cord injury or neurological disorders^1–7^. Hyper-reflexia occurs when the detrusor of the bladder contracts at low volume and causes incontinence^1,2^. Detrusor underactivity has been defined by the *International Continence Society* as a contraction with low strength and/or short duration that causes prolonged or incomplete emptying of the bladder^1,3,7^. Detrusor sphincter dyssynergia (DSD) is a condition that the bladder and external urethral sphincter (EUS) contract together and leads to inefficient voiding with high residual volumes and high intravesical pressures^4–6^. High bladder pressures can cause upper tract deterioration, such as vesico-ureteric reflux, hydronephrosis, renal impairment, and finally end-stage renal disease^7^.

Functional electrical stimulation has been proposed as a means of enhancing bladder voiding and preventing urinary incontinence^8–11^. Sacral anterior root stimulation is represented as the first proposed electrical stimulation system for bladder emptying in spinal cord injury subjects^7,11,12^. However, sacral anterior root stimulation requires a permanent rhizotomy of the sensory dorsal roots to prevent DSD that limits voiding. Destructiveness and irreversibility of the dorsal rhizotomy and sacral denervation could cause the loss of reflex sexual functions and reflex defecation^7^.

Electrical stimulation of the pudendal nerve (PN) has been proposed as an alternative approach to restore urinary function^7^ in individuals with spinal cord injury. The PN carries sensory and motor axons derived from the sacral roots of S2–S4 and innervates regions of the rectal canal, anus, perineum, and external genitalia. It has been shown that the selective frequency-dependent continuous electrical stimulation of the different PN branches including afferent sensory nerve ($2 Hz\le f\le 50$ Hz), cranial urethral sensory nerve ($f\le 5$ Hz), dorsal genital ($f\ge 20$ Hz), and efferent rectal perineal ($f\le 10$ Hz) could evoke bladder contraction individually^13^. It was demonstrated that the voiding efficiency produced by pudendal afferents stimulation was significantly higher than distension-evoked voiding. In another study, it was demonstrated that selective continuous co-stimulation of multiple PN branches of the cat model could evoke larger bladder contraction and provide higher voiding efficiency by selective continuous co-stimulation of multiple PN branches when compared to stimulation of any single PN afferent pathway alone^14^.

It was shown that the voiding efficiency in rats could be improved to $40-51\%$ by the low-intensity electrical stimulation (1–50 Hz) of the proximal end of the unilateral transected sensory branch of the PN^15^. In another study in felines, it was shown that stimulation of the dorsal nerve of the penis at 5–10 Hz inhibited bladder contraction. But, stimulation of the dorsal nerve of the penis at 20–40 Hz could evoke bladder contraction and increase voiding efficiency^16^.

Although continuous pudendal afferent nerve stimulation can generate robust reflex bladder contractions and evoke a sustained high bladder pressure, PN stimulation also activates directly the efferent innervation of the EUS which results in DSD. There have been efforts to use a combination of the low frequency and high frequency stimulation to induce specific lower urinary tract activity and as a therapeutic treatment option for DSD in neurogenic bladder patients. The low frequency continuous stimulation of the afferent PN^17–20^, S2 nerve roots^21,22^, and pelvic nerve^23^ has been used to initiate reflex bladder contraction and voiding. Concurrently with the low frequency stimulation, high-frequency blocking stimulation has been applied to the efferent PN^17–21^, S1 nerve root^22^, and the pelvic nerve^23^ to suppress EUS contractions, and in turn to decrease intraurethral pressure.

Intermittent stimulation of the S2, S3, and S4 anterior roots^7,12^ in humans and PN^24,25^ in cats has also been proposed to overcome the problem of DSD and to improve the voiding efficiency. The post-stimulus method uses the fact that the sphincter muscle consists of fast-twitch fibers and the detrusor muscle of slow-twitch fibers. During pause durations of intermittent stimulation, the sphincter muscle relaxes faster than the detrusor muscle and the bladder empties in spurts. It has been shown that burst stimulation (2–10 pulses at 100–200 Hz) on pudendal nerve trunk at constant continuous frequencies (varied 0.5–100 Hz) could significantly evoke larger bladder responses than continuous stimulation^26^. The bladder responses were evaluated under an isovolumetric condition at bladder volumes up to the occurrence of distension evoked reflex contraction. In another study^27^, it was demonstrated that placement of electrodes near the proximal urethra could evoke bladder contractions with minimal sphincter activation. It was shown that the lower frequency burst-patterned stimuli achieved greater bladder voiding efficiency than other stimulus patterns. Moreover, the effects of different stimulation patterns, applied to the cuff electrode placed around the dorsal genital afferents of the PN, on the bladder contraction were evaluated under isovolumetric conditions^28^. It was shown that the stimulation pattern significantly affected the evoked bladder contraction and no pattern evoked larger bladder contraction than the regular stimulation pattern.

Intraspinal microstimulation (ISMS) has also been explored as an alternative to peripheral nerve stimulation for restoring the bladder function^29–31^. Bladder and urethral pressures evoked by ISMS of the sacral segments S1–S2 were evaluated in neurologically intact anesthetized cats^29^. It was demonstrated that the microstimulation of the specific regions within the spinal cord could generate bladder contraction without increasing the urethral pressure. Microstimulation around the central canal could produce bladder contractions with either no change or a reduction in urethral pressure. It was shown that microstimulation of the S2 lateral ventral horn or ventral funiculus in cats by constant stimulation via a single microelectrode could generate high bladder contraction with small urethral contraction^30^. However, this stimulation did not evoke complete voiding. In another study^31^ in cat models, it was reported that ISMS of the sacral parasympathetic nucleus at the S2 segment or of the adjacent ventrolateral white matter produced bladder contraction but not sufficient for voiding, while stimulation of the dorsal gray commissure or of immediately dorsal to dorsal gray commissure at the S1 segment by a constant frequency of 20 Hz produced strong bladder contractions as well as strong EUS relaxation.

All the aforementioned methods are based on the open-loop control manner. A closed-loop control has been proposed for control of bladder function while the intravesical pressure (IVP) has been employed as the feedback information^32,33^. Two pressure thresholds were defined to trigger the low-frequency continuous PN stimulation and intermittent high-frequency pudendal afferent stimulation. However, the method resulted in poor emptying the bladder ($25.0\%$). In another study, a closed-loop control has also been proposed for stimulating the pelvic nerves in anesthetized rats to evoke bladder contractions^33^. First, a bladder state classification based on a defined threshold of IVP was performed to detect the stimulation outcomes (i.e., voiding or non-voiding). Then, the current amplitude was adjusted based on the bladder state. Classification accuracy of $70.5\%$ and voiding efficiency of $75.7\pm 3.0\%$ were reported.

In this paper, we propose a closed-loop control of ISMS for efficient bladder voiding. The strategy is based on multielectrode ISMS with combined pulse-amplitude and pulse-frequency modulation; we call it hybrid amplitude-frequency modulation. Unlike the previous works, the bladder volume is used as feedback information. The dynamic response of the bladder pressure and volume generated by ISMS paradigm are also evaluated. It is demonstrated that the proposed strategy significantly improves the bladder voiding efficiency with respect to the previous works.

## Methods

### Animal preparation and surgery

The experiments were conducted on twenty-nine intact adult male Wistar rats (250–350 g). All surgical procedures and experimental paradigms involving animal models described in this paper were approved by the Animal Care and Ethics Committee of Iran Neural Technology Research Centre, Iran University of Science and Technology. The experiments were performed in accordance with the recommendations and relevant guidelines. Moreover, the study was carried out in compliance with the ARRIVE guidelines.

**Figure 1:**
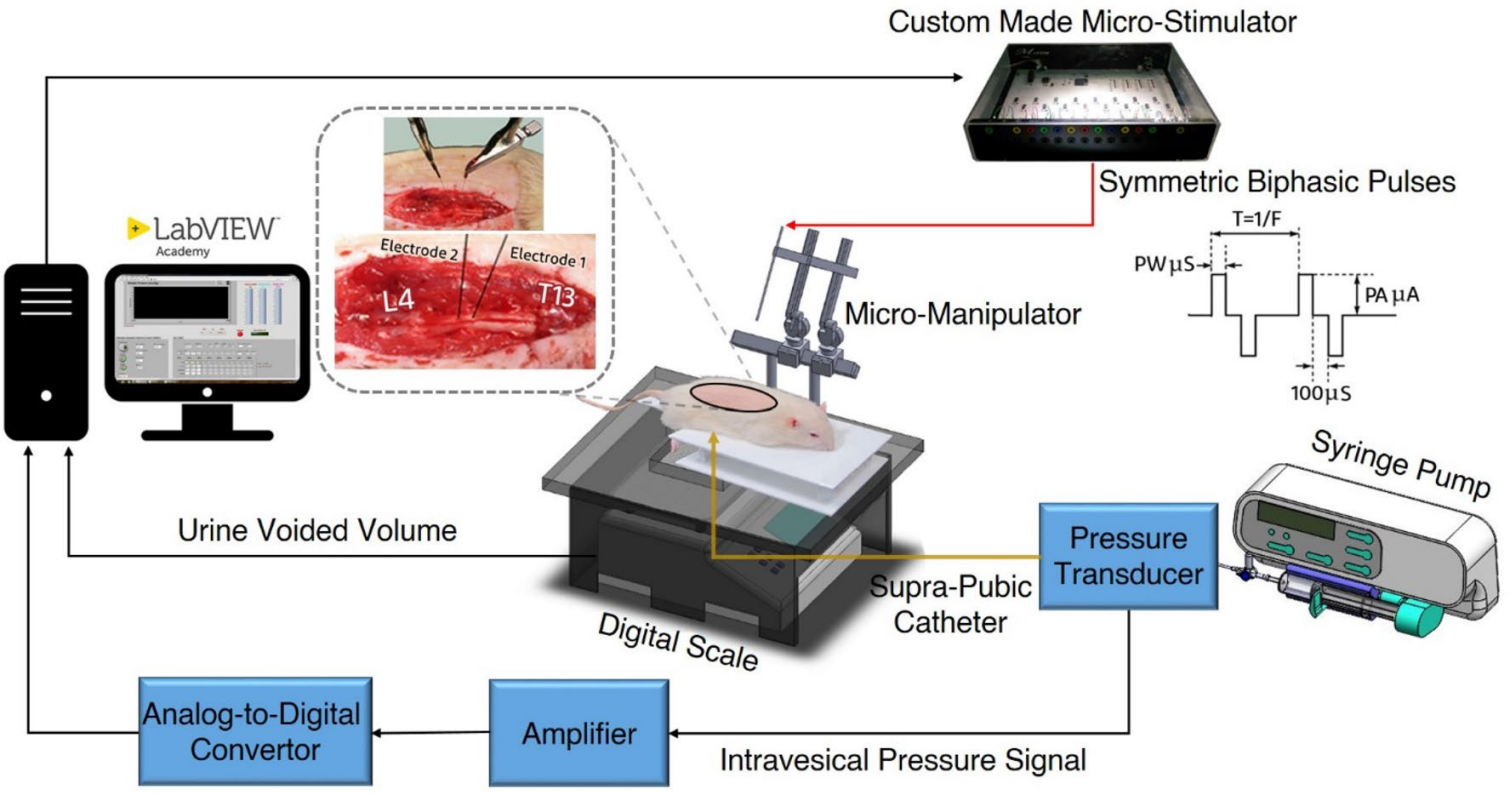
Schematic representation of the experimental setup for closed-loop control of bladder voiding using intraspinal microstimulation (ISMS). A supra-pubic catheter placed in the bladder is connected to a pressure transducer and an infusion pump via a 3-way connector. The digital scale is positioned under the rat to measure the voided volume. By continuously subtracting the voided volume from the filled volume, the residual volume can be calculated. Two micro-electrodes are inserted into the S1 and S2 spinal cord segments for ISMS. The closed-loop control system automatically adjusts the stimulation intensity through online monitoring the bladder volume to provide efficient bladder voiding. Schematic figures showing syringe pump, micro-manipulator, and digital scale were prepared using an open source computer aided design software (Blender 2.8, Blender Foundation, Amsterdam, Netherlands).

The animals were deeply anesthetized by intraperitoneal administration of urethane (1.5 g/kg) and supplemented as needed. Urethane is the preferred anesthetic agent to use in urodynamic studies because the micturition reflex is preserved^34^. An incision was made at the ventral midline abdominal to expose the urinary bladder. Then, a polyethylene (PE) tube 50 (0.5 mm inner diameter, 0.9 mm outer diameter, ADInstruments Ltd, Australia) was inserted into the bladder wall through the dome and secured with a 6–0 silk suture. The abdominal wall was closed in layers with 3–0 silk suture. The catheter was connected to a pressure transducer (NovaTrans Transducer Systems, MX860, Smiths Medical) and an infusion pump (SN-50C6, Sino Medical-Device Technology Co., Ltd.) via a 3-way connector. Then, a partial laminectomy was performed on the lumbar vertebrae (L1 to L2) to expose the L6-S3 segments^35^ and the dura mater over these segments was opened longitudinally. Warm mineral oil was applied to the exposed spinal cord to prevent desiccation during the experiment. The rats were positioned in a modified stereotaxic setup (SR-6R, Narishige Group Product), which allows the hind limbs to hang free while the head and spinal vertebrae (T13 and L4) are clamped to the frame. The infused volume was computed by multiplying the infusion rate by filling duration in each experiment trial. A digital scale (GF-300, A & D Instrument, Ltd., UK) with serial port communication was positioned under the rat to measure the voided volume. By continuously subtracting the voided volume from the injected volume, the residual volume could be calculated. The bladder pressure was amplified (900x) and sampled at 50 Hz with a 12-bit analog-to-digital converter (Advantech PCI-1711L I/O card). The experimental setup is illustrated in Fig. 1.

### Stimulation electrode implantation

The stimulating microelectrodes were implanted in the lumbosacral region of the spinal cord. The electrodes were made from epoxylite-insulated tungsten with shank diameter 125-μm, 15°–20° tapered tip, and rounded tip with ~ 3-micron diameter at the tip (FHC Inc., Bowdoin, ME USA). To control the three-dimensional positioning of electrodes in the lumbosacral region of the spinal cord, each microelectrode was mounted on a micromanipulator (SM-25A, Narishige Group Product). An electrode was placed on the longissimus muscle of the spinal cord as the reference electrode for all stimulation electrodes.

To determine the best stimulating position in the lumbar spinal cord for bladder contraction and voiding, the microelectrode was advanced dorsoventrally through the spinal cord. The microelectrode delivered a train of constant current (80–100 μA) biphasic pulses at 20 Hz and 80 μs pulse width. Then, the electrode was removed and reinserted in 400 μm increments rostrocaudally from L6 to S3 segment, and/or mediolaterally to an adjacent location where the best dorsoventral position was determined, and the spinal cord was stimulated. The stimulating locations with the highest voiding efficiency were selected (i.e., S1–S2). The depth of the electrode penetration was estimated using the calibrated bars of the micromanipulator.

**Figure 2:**
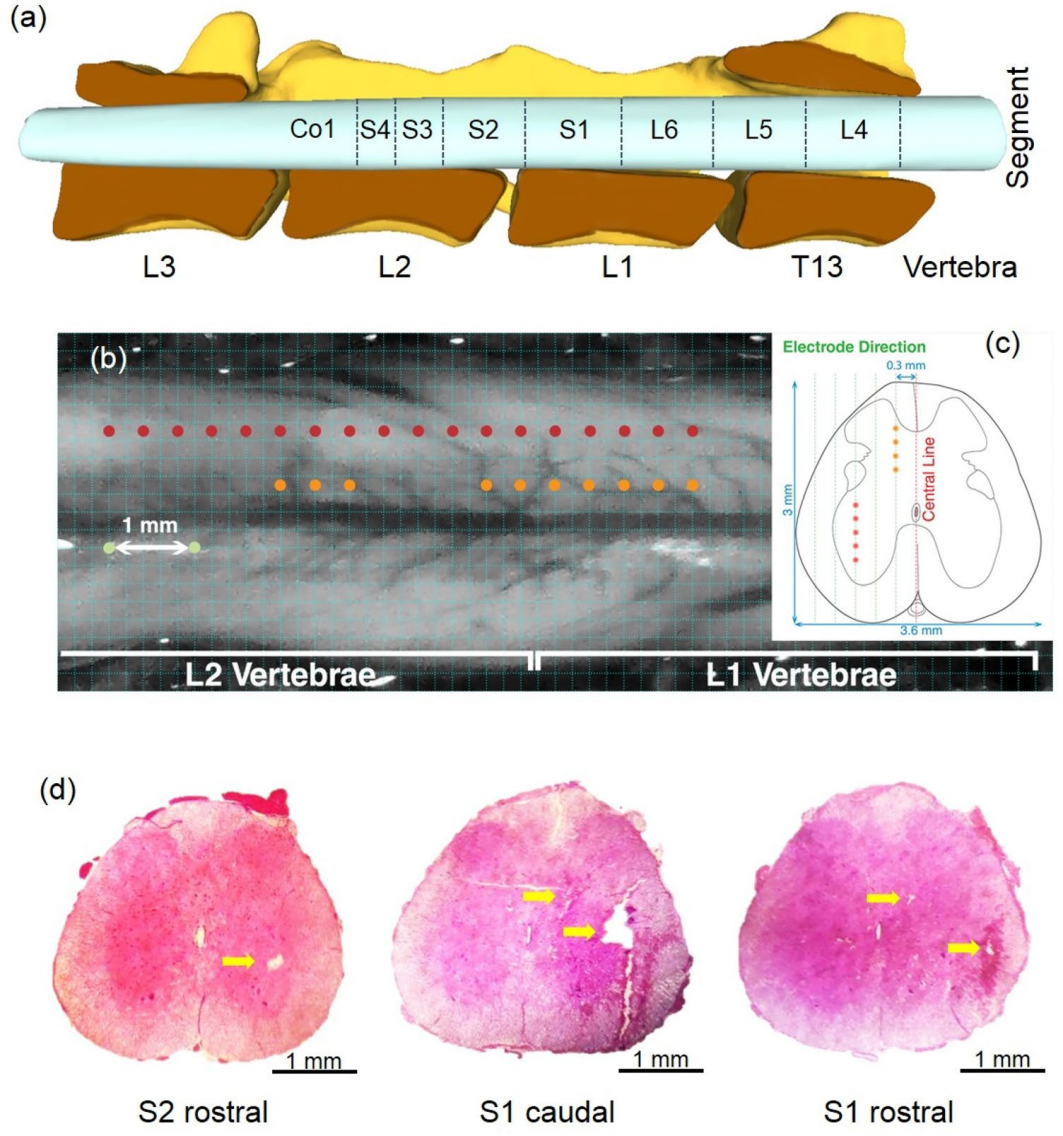
Overview of stimulation microelectrode sites within the spinal cord. (**a**) Schematic figure showing the position of the spinal segments with respect to the spinal vertebrae. (**b**) Dorsal view of the surface of the spinal cord showing the positions of implanted electrodes across subjects. A 200-µm grid was superimposed on a digital photograph of the exposed spinal cord. The orange and red dots illustrate the positions of the implanted microelectrodes across subjects. (**c**) A cross-sectional view of the rat spinal cord showing the locations of the microelectrode stimulation at the lateral ventral horn and dorsal gray commissure. (**d**) Different histological cross-sections are showing different positions of the implanted electrodes. Schematic figure showing the position of the spinal segments (**a**) was prepared using Blender 2.8 (Blender Foundation, Amsterdam, Netherlands).

Figure 2 shows the dorsal and the cross-sectional view of the spinal cord showing the microelectrode stimulation sites. A digital color photograph of the exposed spinal cord was taken using a compact digital camera. The image was carried to Adobe Photoshop (Berkeley, CA: Peachpit Press, 2004. Print.), where it was cropped, resized, and converted to black and white theme, and enhanced with a Darker filter. Then a 200-µm grid was superimposed using Matlab custom-made function. The electrode tip positions were shown with dots (Fig. 2b). To illustrate the depth of the penetrated microelectrode tips, the cross-sectional view of the rat spinal cord was shown in Fig. 2c. The location of the target area that efficiently activated bladder contraction slightly differed from a rat to another.

In a series of experiments, at the end of the stimulation, electrolytic lesions were made in the spinal cord at the microelectrode positions by delivering direct current at 20 µA for 10 s. Then, the microelectrode was removed, and the spinal cord was fixed in situ by immersion in a 10% formaldehyde solution. Finally, the animals were euthanized. The spinal segments of interest were excised and the 50-µm thick sections were sliced, stained with Hematoxylin and Eosin (H&E), and mounted on the slides for histological verification of the stimulating electrode track. The sections were examined using an Olympus BX-41 and an Olympus 5 Mega pixel DP10 digital camera (Olympus Corporation, Tokyo, Japan). Figure 2d shows typical histological analysis showing the microelectrode sites in the dorsal gray commissure and lateral ventral horn.

### Voiding trials

During each trial of experiment the bladder was filled at a rate of 20 mL/h until the bladder volume reached approximately 90% of the threshold volume (${V}_{th}$). The threshold volume (${V}_{th}$) was defined as the bladder volume at which the distension-evoked bladder contractions occurred. During each session of experiment on a rat, the ${V}_{th}$ was determined by filling the bladder with saline at the physiologic infusion rate of 3–6 ml/h^1^. At the end of each trial of experiment, the bladder was first manually emptied with a syringe. A rest interval of about 20 min between voiding trials was considered for bladder relaxation. During each session of experiment, 4–6 trials were conducted and the duration of each trial was between 3 and 7 min.

### Stimulation protocols

A custom-made 16-channel computer-based micro-stimulator was used to stimulate the spinal cord. The stimulation consisted of charge-balanced, cathodic-first symmetric biphasic current-controlled pulses with a fixed pulse-width of 80 µs, inter-phase delay of 100 µs, and amplitude of 40–150 μA. This resulted in electrical charges of 0.032–12 nC/phase and, taking into account the geometry of the electrode contact, charge densities of 0.97–363 µC/cm^2^ per phase. Those values are below the ‘safe limits’ known from both animal studies and patient research^36^. Voiding efficiency was evaluated by different intermittent stimulation paradigms:

- Single-electrode pulse amplitude modulation (PAM) with a frequency of 20 Hz at either 33% or 50% duty cycle.
- Single-electrode stimulation with constant pulse amplitude at a frequency of 20 Hz with 33% duty cycle.
- Single-electrode stimulation with hybrid amplitude-frequency modulation at 33% duty cycle.
- Two-electrode pulse amplitude modulation at a frequency of 20 Hz at either 33% or 50% duty cycle under either synchronous or asynchronous mode of stimulation (see Fig. 4).
- Two-electrode asynchronous stimulation with hybrid amplitude-frequency modulation at 33% duty cycle.

### Closed-loop ISMS control strategy

**Figure 3:**
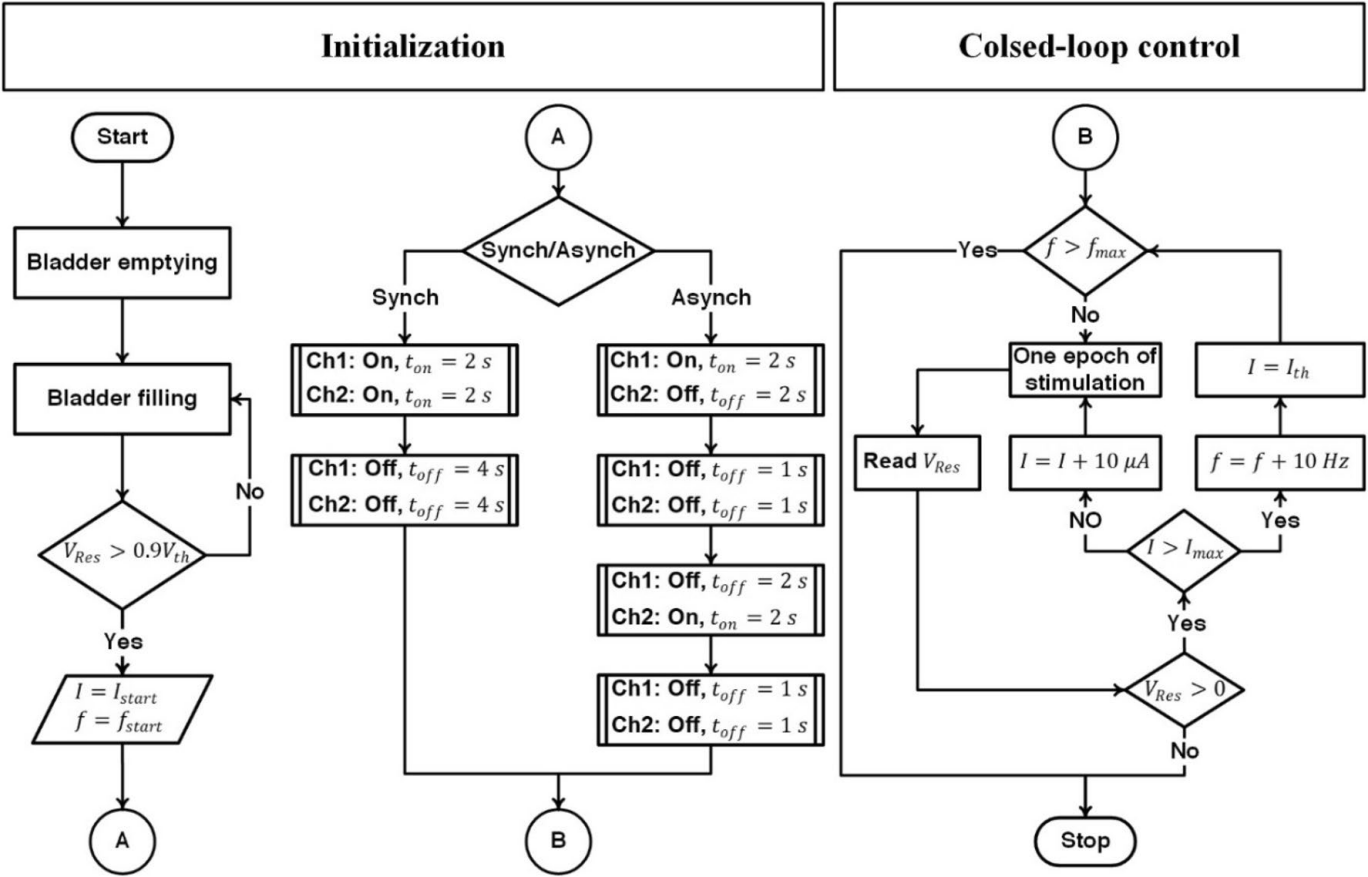
Representative diagram of the proposed closed-loop control system. The proposed method is based on the two-electrode ISMS using hybrid pulse-amplitude and pulse-frequency modulation. ${I}_{start}$ is the initial current amplitude for 20 Hz and ${I}_{th}$ is for 30 Hz stimulation, ${I}_{max}$ is the maximum allowable current amplitude, ${f}_{start}$ is the initial stimulation frequency, and ${f}_{max}$ is the maximum allowable stimulation frequency. To initiate the closed-loop control, the bladder was first manually emptied with a syringe and then filled until the volume reached approximately $90\%$ of ${V}_{th}$. The pulse amplitude was set to ${I}_{start}$, frequency to ${f}_{start}$, and the mode of stimulation to synchronous or asynchronous. After initialization, the first epoch of the closed-loop control was started. One epoch of stimulation consists of either 33% duty cycle (2 s ON, 4 s OFF) or 50% duty cycle (2 s ON, 2 s OFF). At the end of the stimulation epoch, the stimulation parameters were adjusted according to the bladder residual volume. The execution of the subsequent epochs of stimulation was dependent on the residual volume and ${f}_{max}$. If there is volume left in the bladder after stimulation, the current amplitude was incremented by 10 μA steps for each subsequent epoch. If post-stimulation bladder volume level remains higher than zero and the current amplitude reaches ${I}_{max}$, the frequency is incremented by 10 Hz and the pulse amplitude is set to ${I}_{th}$ and the closed-loop control is continuing by the new initial values. The closed-loop process continues until the residual volume reaches zero or the frequency to ${f}_{max}$.

**Figure 4:**
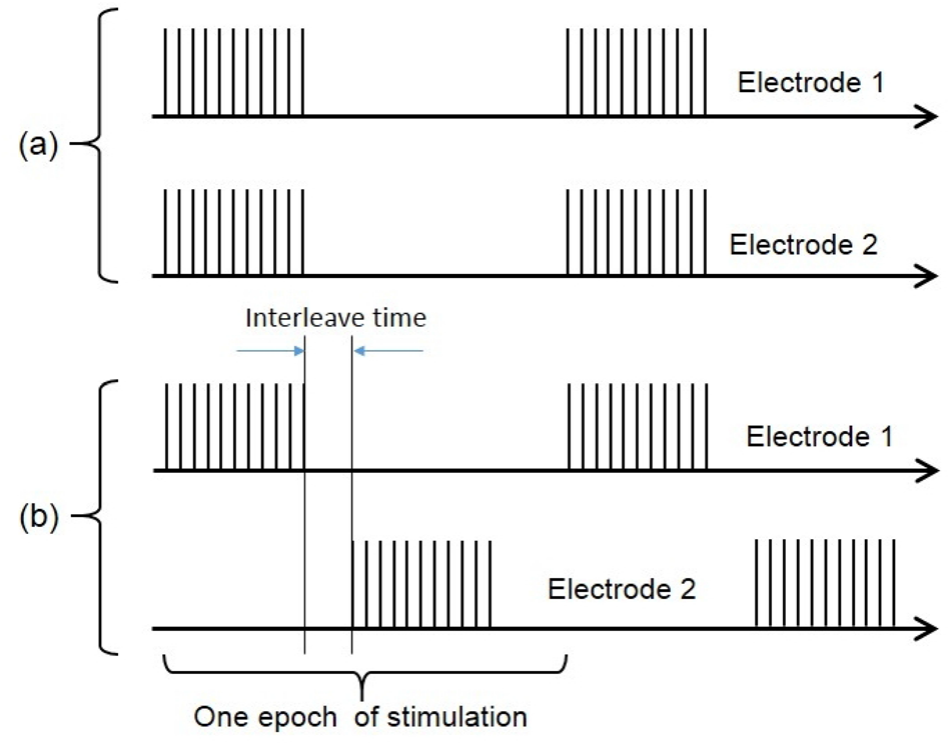
Two modes of the two-electrode ISMS. (**a**) Synchronous mode. In synchronous mode, stimulus pulses were delivered to the two electrodes simultaneously with interleave time 0. (**b**) Asynchronous mode. In asynchronous mode, stimulus pulses were delivered to the two electrodes with an interleave time.

A representative diagram of the proposed closed-loop control strategy is shown in Fig. 3. The proposed method is based on the two-electrode ISMS using combined pulse-amplitude and pulse-frequency modulation. During two-electrode stimulation, two modes of stimulation have been used to stimulate the spinal cord: synchronous and asynchronous. In synchronous mode, stimulus pulses were delivered to the two electrodes simultaneously with interleave time 0 (Fig. 4), while in asynchronous mode, stimulus pulses were delivered to the two electrodes with interleave time either 3 s when the duty cycle is 33% (2 s ON, 4 s OFF) or 2 s when the duty cycle is 50% (2 s ON, 2 s OFF).

To initiate the closed-loop control, the bladder was first manually emptied with a syringe and then filled until the volume reached approximately $90\%$ of ${V}_{th}$. The pulse amplitude was set to ${I}_{start}$, frequency to ${f}_{start}$, and the mode of stimulation to synchronous or asynchronous. After initialization, the first epoch of the closed-loop control was started. One epoch of stimulation consists of either 33% duty cycle or 50% duty cycle (Fig. 4). At the end of the stimulation epoch, the stimulation parameters were adjusted according to the bladder residual volume. The execution of the subsequent epochs of stimulation was dependent on the residual volume and ${f}_{max}$. If there is volume left in the bladder after stimulation, the current amplitude was incremented by 10 μA steps for each subsequent epoch. If post-stimulation bladder volume level remains higher than zero and the current amplitude reaches ${I}_{max}$, the frequency is incremented by 10 Hz and the pulse amplitude is set to ${I}_{th}$ and the closed-loop control is continuing by the new initial values. The closed-loop process continues until the residual volume reaches zero or the frequency to ${f}_{max}$.

The closed-loop ISMS system uses a custom-designed LabVIEW software for online monitoring of the bladder volume and generating the stimulus pulses with the sampling period of 20 ms for control updates.

### Stimulation parameter setting

A series of experiments were conducted to evaluate the effect of the current amplitude and stimulation frequency and to determine the initial current amplitude (${I}_{start}$), the maximum allowable current amplitude (${I}_{max}$), the initial stimulation frequency (${f}_{start}$), and the maximum allowable stimulation frequency (${f}_{max}$) for the closed-loop control system. To determine ${I}_{start}$ and ${I}_{max}$, the stimulation was started with an amplitude of 10 μA. Then, the current amplitude was incremented with 10 μA for each subsequent epoch until the amplitude reached ${I}_{max}$ The maximum intensity was set to avoid hind limb movements and body tremors. The lowest current amplitude was selected such that the relative changes in voiding volume for the first two consecutive stimulation to be significant. This approach was repeated for each frequency step to select the lowest current amplitude (i.e., ${I}_{start}$ is the initial current amplitude for 20 Hz and ${I}_{th}$ is for 30 Hz stimulation). To select the most effective stimulation frequency, the spinal cord was stimulated under an isovolumetric condition at a low volume state ($0.25{V}_{th}$) with different stimulation frequencies.

### Data analysis methods

Cystometry parameters were used to evaluate the performance of the proposed closed-loop ISMS control strategy for control of bladder voiding. Cystometry parameters included intravesical pressure (IVP), peak pressure, change in intravesical pressures during one epoch of stimulation ($\Delta P$), urine voided volume, residual volume (expressed as the percentage of the bladder volume during 1-s prior to the first epoch of stimulation), and voiding efficiency. The peak pressure was defined as the maximum pressure during the stimulation epoch. A 1-s period prior to each stimulation epoch was used to define the baseline pressure to calculate the change in intravesical pressures during stimulation. The urine voided volume (UVR) and residual volume (RV) were measured to calculate the voiding efficiency (VE) as follows

1 $$\mathrm{VE }\left(\mathrm{\%}\right)=\frac{\mathrm{UVR}}{\mathrm{RV}+\mathrm{UVR}} \times 100$$

For normality testing, either the Shapiro–Wilk test or the Anderson–Darling test and for homogeneity of variance, Levene's test was used. Analysis of variance (ANOVA) was used to compare the voiding parameters under different conditions. For all analyses, $p<0.05$ was considered for a statistically significant difference.

## Results

### Effects of the current amplitude

**Figure 5:**
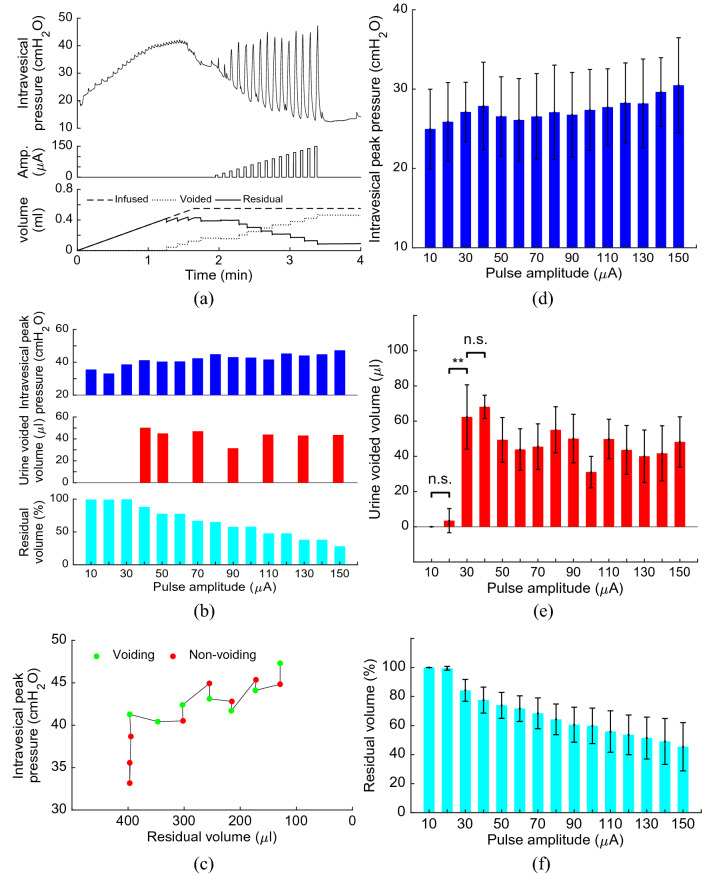
Effect of current amplitude on bladder pressure and voiding. An example of the intravesical pressure and volume evoked by ISMS (Rat 3, trial 1) with the frequency stimulation of 20 Hz and 33% duty cycle (**a**–**c**). The electrode was implanted into the lateral ventral horn of the S2 spinal segment. The epoch of stimulation was started with 10 μA. Then, the current amplitude was incremented with 10 μA for each subsequent epoch. The average of the bladder response over 11 trials of experiment on 3 rats (Rat 1-Rat 3) using single-electrode ISMS with step increases in the stimulation amplitude (**d**–**f**). ** indicates statistical significance ($p<0.01$) and n.s. not statistically significant.

Figure 5 shows an example of the intravesical pressure and volume evoked by ISMS with the frequency stimulation of 20 Hz and 33% duty cycle. The epoch of stimulation was started with 10 μA. Then, the current amplitude was incremented with 10 μA for each subsequent epoch until the amplitude reached ${I}_{max}$. It is observed that, for this trial of experiment, voiding was initiated upon the intravesical peak pressure reached 41.28 cmH_2_O which was evoked by 40 μA stimulation. Although, the intravesical peak pressure was monotonically increased by increasing the stimulation amplitude, but increasing the intravesical peak pressure has not caused the bladder voiding. This is because the voiding is not only depend on the pressure but also on the bladder volume. Figure 5c shows the volume-pressure diagram during stimulations with step increase in the amplitude. It can be seen that the 90 μA stimulation provides higher peak pressure than 80 μA stimulation, but will not cause voiding. This may be due to the bladder volume. The bladder volume during 80 μA stimulation is higher than that during 90 μA stimulation.

The average of the bladder response evoked by step increases in stimulation amplitude is shown in Fig. 5d–f. It can be seen that increasing the stimulation amplitude caused the increase of the intravesical peak pressure in a monotonic manner. However, an increase in the pressure did not always result in voiding. This indicates that the bladder pressure cannot be used as the only feedback information for the closed-loop control. The results show that the voiding outcomes produced by 10 μA and 20 μA are not significantly difference ($p=1,$ two-way ANOVA), but 30 μA stimulation would be able to produce voiding that is significantly higher than that produced by 20 μA ($p=0.0078,$ two-way ANOVA). The 40 μA stimulation produced voiding that is higher than 30 μA stimulation, but it is not significant ($p=0.6533$, two-way ANOVA). Since there is no significant difference between outcomes of 30 μA and 40 μA, the current amplitude of 40 μA stimulation was selected as the ${I}_{start}$ during the closed-loop control of the bladder voiding.

### Effects of the stimulation pattern

**Figure 6:**
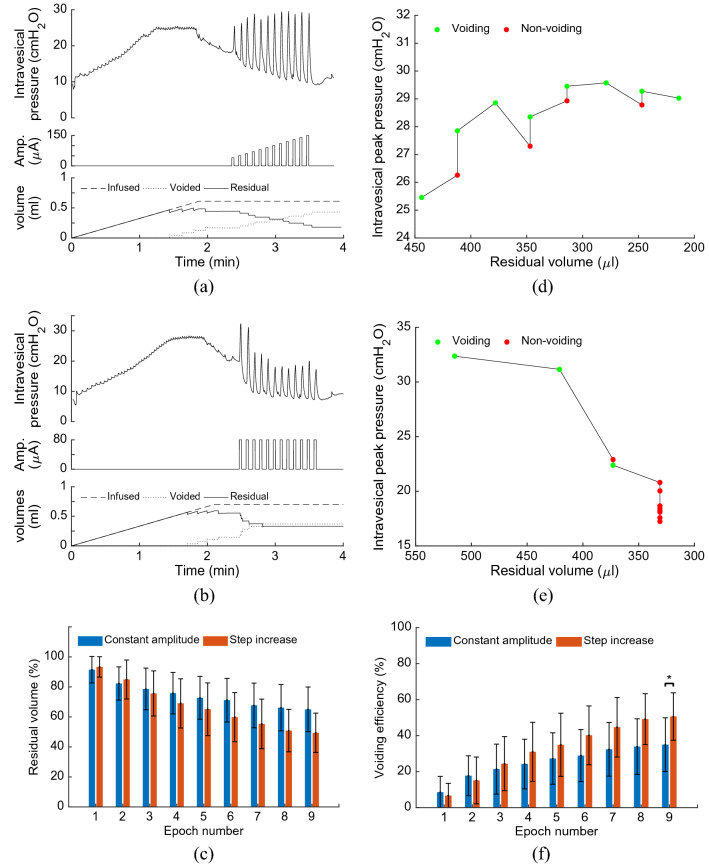
Effect of stimulation pattern on bladder response. An example of the bladder response to the stimulation pattern with step increase in amplitude (**a**,**d**) and stimulation pattern with fixed pulse amplitude (**b**,**e**). The average values of the residual volume (**c**) and the cumulative voiding efficiency (**f**) over five rats (Rat 4–Rat 8) after each epoch of stimulation for both patterns (*$p<0.05$).

Figure 6 shows an example of the bladder responses to the stimulation pattern with a fixed pulse amplitude and the stimulation pattern with step increase in amplitude. During stimulation pattern with step increase, the epoch of stimulation was started with 40 μA. Then, the current amplitude was incremented with 10 μA for each subsequent epoch (Fig. 6a). During the stimulation pattern with fixed amplitude, the pulse amplitude was kept constant at 80 μA (Fig. 6b). It can be seen that the peak pressure monotonically increases by increasing the pulse amplitude during the stimulation pattern with step increase in amplitude (Fig. 6a,d), but during the stimulation pattern with constant amplitude, the maximum pressure is achieved for the first and the second epoch of stimulation, but for the subsequent of epochs, the pressure begins to decrease (Fig. 6b,e).

Figure 6c shows the average of bladder residual volume over five rats after each epoch of stimulation for both patterns. After five epochs of stimulation, which the pulse amplitude reaches 80 μA during stimulation pattern with step increase in amplitude, the voiding efficiencies are $27.3\pm 14\%$ and $34.9\pm 17\%$, for the stimulation pattern with fixed amplitude and stimulation pattern with step increase, respectively. The results show that, after nine epochs of stimulation in which the total injected charge of both patterns is equal, the pattern with step increase provides significantly higher voiding efficiency than the pattern with fixed amplitude ($p=0.0286,$ one-way ANOVA). The average voiding efficiency obtained using both stimulation patterns is shown in Fig. 6f.

### Effect of the stimulation frequency

**Figure 7:**
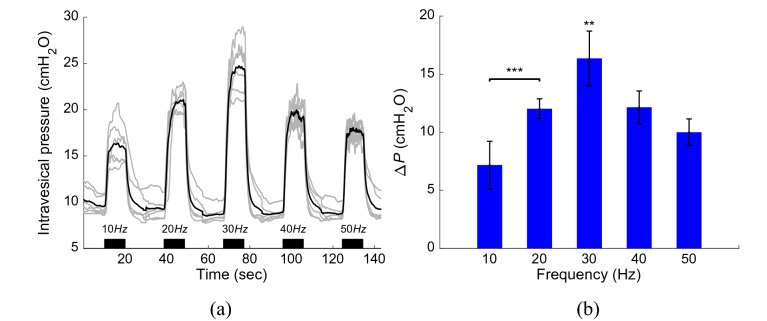
Effect of the stimulation frequency on the intravesical pressure under the isovolumetric condition in five rats (Rat 9- Rat 13). (**a**) Intravesical pressure evoked by ISMS with different frequencies. The stimulation duration and inter-stimulation interval were 10 s and 20 s, respectively. (**b**) The average of $$\Delta P$$ evoked by ISMS with different frequencies. Stimulating at a frequency of 30 Hz generated significant intravesical pressure compared to the stimulating at frequencies 10, 20, 40, and 50 Hz ($$**p<0.01, ***p<0.001$$, two-way ANOVA).

Figure 7 shows the effects of the stimulation frequency on the intravesical pressure under the isovolumetric condition at a low volume state ($$0.25{V}_{th}$$) in five rats. The stimulation duration and inter-stimulation interval were set to 10 s and 20 s, respectively. The results show that stimulating at a frequency of 30 Hz generated significant intravesical pressure ($$p<0.01$$) compared to the stimulating at frequencies 10, 20, 40, and 50 Hz. An interesting observation is that stimulating at higher frequency (40 or 50 Hz) reduced the intravesical pressure. This decrease in intravesical pressure may be due to the inhibitory effect of the interneurons during high frequency stimulation. Using a computational model of the spinal neural network, Mcgee and Grill^37^ have shown that during bursts of high frequency stimulation, the interneurons failed to increase their firing rate to match the input, and prevented the sacral parasympathetic nucleus from firing more than once. Moreover, the intravesical pressure generated by 20 Hz is significantly higher than that by 10 Hz ($$p<0.001)$$. In this study, the frequencies of 20 Hz and 30 Hz were selected as the $${f}_{start}$$ and $${f}_{max}$$, respectively, for the closed-loop control. Since, the bladder volume is high at the start of the closed-loop control, 20 Hz stimulation was selected to initiate the control.

### Closed-loop control

#### Single-electrode versus two-electrode closed-loop control

In this section, the effects of multielectrode stimulation on voiding efficiency during closed-loop control of ISMS with pulse amplitude modulation (PAM) are investigated and the results are compared with the single-electrode closed-loop control. During multielectrode closed-loop control, two modes of stimulation, synchronous and asynchronous stimulation, were considered.

**Figure 8:**
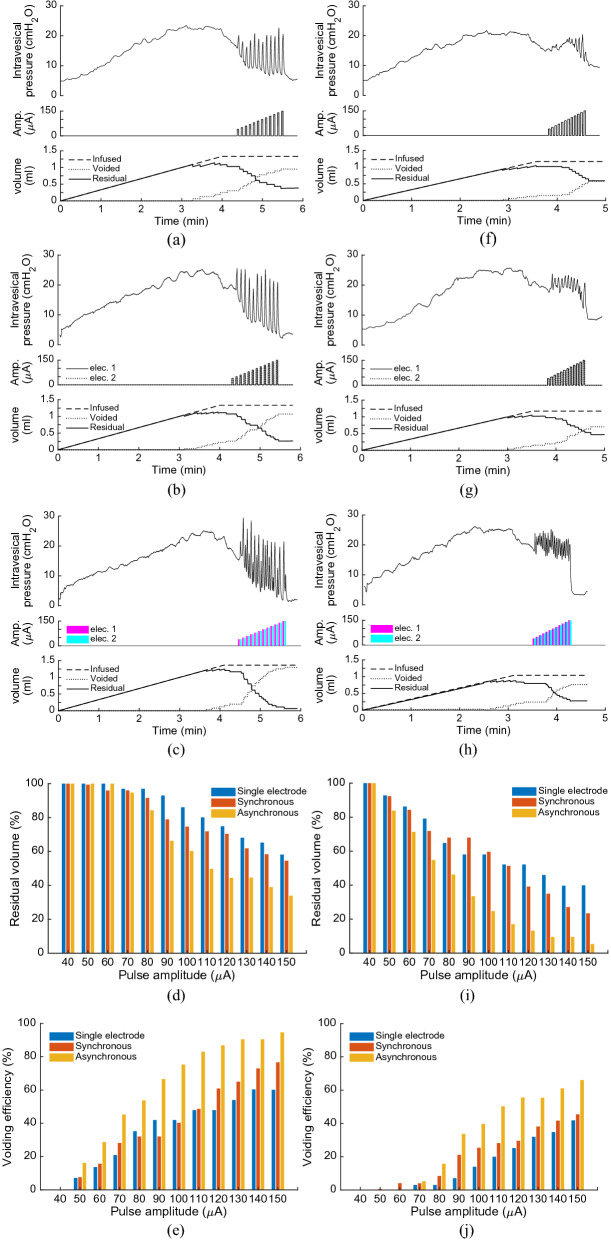
An example of the bladder response during single-electrode and multielectrode closed-loop control of ISMS with pulse amplitude modulation (PAM) at duty cycle of 33% as well as at duty cycle of 50% (Rat 16). Single-electrode stimulation (**a**), two-electrode synchronous stimulation (**b**), and two-electrode asynchronous stimulation (**c**) with duty cycle of 33% and 20 Hz. Residual volume (**d**) and voiding efficiency (**e**) during single-electrode and multielectrode closed-loop control of ISMS with PAM at duty cycle of 33%. (**f**–**j**) The same information as in (**a**–**e**) when stimulation with duty cycle of 55% is applied.

Figure 8 shows an example of the bladder response during single-electrode and multielectrode closed-loop control of ISMS with PAM at duty cycle of 33% as well as at duty cycle of 50%. The results for these trials of experiment show that the multielectrode stimulations provide higher voiding efficiency than single-electrode stimulation for both 33% and 50% duty cycle. The voiding efficiencies achieved with 33% duty cycle of stimulation are $60.12\%$, $76.63\%$, and $94.66\%$ using single-electrode, two-electrode synchronous, and two-electrode asynchronous closed-loop control, respectively, while the efficiencies obtained with 50% duty cycle are $41.85\%$, $45.45\%$, and $66.10\%$. It is observed that the stimulations with 50% duty cycle provide a sustained elevation of bladder pressure. However, this sustained bladder pressure would not result in a higher voiding efficiency than the stimulations with 33% duty cycle. Moreover, asynchronous stimulation achieves higher efficiency than synchronous stimulation. A possible explanation for this observation is that the spatial sum of two vector fields produced by the synchronous stimulation at two sides can produce a net field that is greater than either of its parts. This may result in direct activation of pudendal motoneurons in the ventral horns which will cause an increase in urethra pressure^29^ and, consequently, a decrease in voiding.

**Figure 9:**
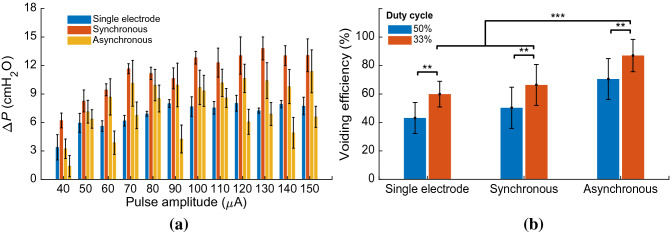
Comparison of single-electrode and multielectrode closed-loop control of ISMS. In this comparison, the stimulation with PAM strategy and 33% duty cycle was used during single-electrode and multielectrode closed-loop control. The average values of $$\Delta P$$ (**a**) and voiding efficiency (**b**) over ten rats (Rat 14–Rat 23) using single-electrode and multielectrode closed-loop control of ISMS ($$**p<0.01, ***p<0.001$$, two-way ANOVA). The third and fourth columns in (**a**) show the $$\Delta P$$ that generated by the first and second electrodes, respectively, during two-electrode asynchronous stimulation. Two-electrode synchronous stimulation provides higher intravesical peak pressure than single-electrode and two-electrode asynchronous stimulation.

Figure 9 shows the average of the bladder response over ten rats using single-electrode and multielectrode closed-loop control of ISMS with PAM. It is observed that two-electrode synchronous stimulation provides higher intravesical peak pressure than single-electrode and two-electrode asynchronous stimulation (Fig. 9a). However, this higher pressure would not result in higher voiding efficiency. The third and fourth columns in Fig. 9a show the $\Delta P$ that generated by the first and second electrode, respectively, during two-electrode asynchronous stimulation. The average values of voiding efficiency with 33% duty cycle are $57.9\pm 12\%$, $70.3\pm 15\%$, $87.0\pm 11\%$, using single-electrode, two-electrode synchronous, and two-electrode asynchronous stimulation, respectively, while with 55% duty cycle are $42.1\pm 13\%$, $54.1\pm 12\%$, and $70.2\pm 14\%$. The results indicate that the two-electrode stimulation significantly improves the voiding efficiency compared to the single-electrode stimulation ($p=5.6779\times {10}^{-4},$ N-way ANOVA) and two-electrode asynchronous compared to the two-electrode synchronous stimulation ($p=3.6412\times {10}^{-5},$ two-way ANOVA). Moreover, the results show that the stimulation with 33% duty cycle significantly improves the voiding efficiency compared to the stimulation with 50% duty cycle ($p=5.1236\times {10}^{-6},$ N-way ANOVA).

### Hybrid closed-loop control

**Figure 10:**
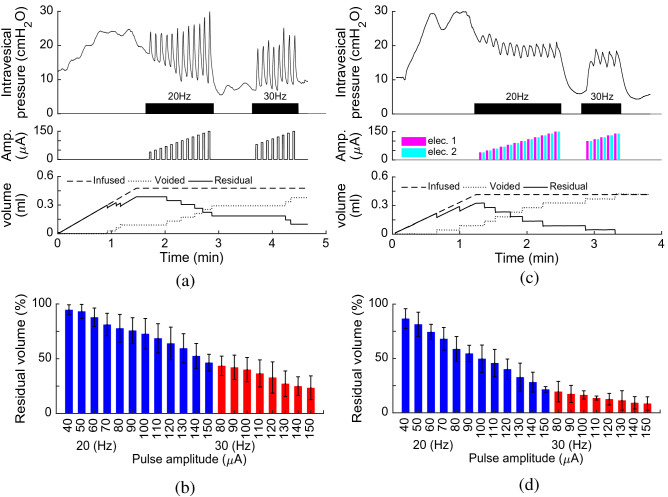
A typical trial of hybrid closed-loop control of ISMS using single-electrode as well as two-electrode stimulation with duty cycle of 33%. (**a**) Single-electrode. (**b**) The average of the residual volume over 6 rats (Rat 24–Rat 29) during single-electrode hybrid closed-loop control for each epoch of stimulation. (**c**) Two-electrode asynchronous stimulation. (**d**) The average of the residual volume over 6 rats (Rat 24–Rat 29) during two-electrode hybrid closed-loop control.

Figure 10 shows a typical trial of hybrid closed-loop control of ISMS using single-electrode as well as two-electrode stimulation. For this trial of experiment, the voiding efficiencies are $73.3\%$ and $100\%$ using single-electrode and two-electrode stimulation, respectively. The stimulations are taken 170 s and 131 s during single-electrode and two-electrode closed-loop control of ISMS, respectively. It should be noted that the stimulation is terminated during hybrid stimulation if the residual volume reaches zero or the current amplitude and stimulation frequency reaches ${I}_{max}$ and ${f}_{max}$, respectively.

**Figure 11:**
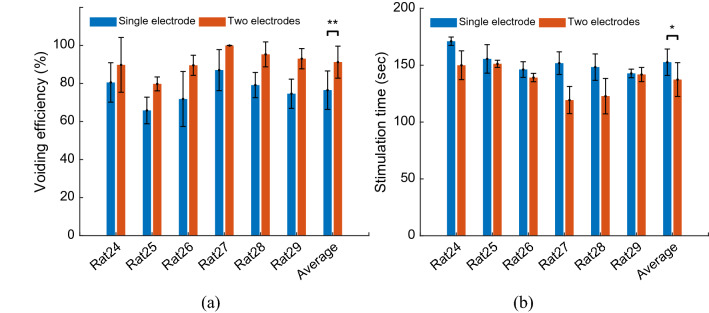
Average bladder response for each rat during closed-loop control of ISMS with hybrid asynchronous stimulation and 33% duty cycle. The average of voiding efficiency (**a**) and the average of stimulation time taken (**b**) for each rat during closed-loop control of ISMS with hybrid asynchronous stimulation and 33% duty cycle ($$*p<0.05, **p<0.01$$, two-way ANOVA).

The average of voiding efficiency for each rat using closed-loop control of ISMS with hybrid stimulation is shown In Fig. 11a. The average voiding efficiency over all rats is $76.6\pm 10.1\%$ and $91.28\pm 8.4\%$ using single-electrode and two-electrode stimulation, respectively. The results of the two-way ANOVA show that two-electrode stimulation significantly improves the voiding efficiency compared to the single-electrode stimulation ($p=0.0013$). There is no significant difference between the voiding efficiencies of the rats ($p=0.0873$) and there is no interaction between the column factors (i.e., single-electrode and two-electrode stimulation) and the rats ($p=0.9740$).

The average stimulation time for each rat during closed-loop control of ISMS with hybrid stimulation is shown in Fig. 11b. The average of the stimulation time over all rats are $155\pm 13$ s and $138\pm 6$ s using single-electrode and two-electrode stimulation, respectively. The results show that the simulation time taken during two-electrode asynchronous stimulation is significantly shorter than that single electrode stimulation ($p=0.0488,$ two-way ANOVA). There is no significant difference between the simulation times of the rats ($p=0.0941,$ two-way ANOVA).

**Figure 12:**
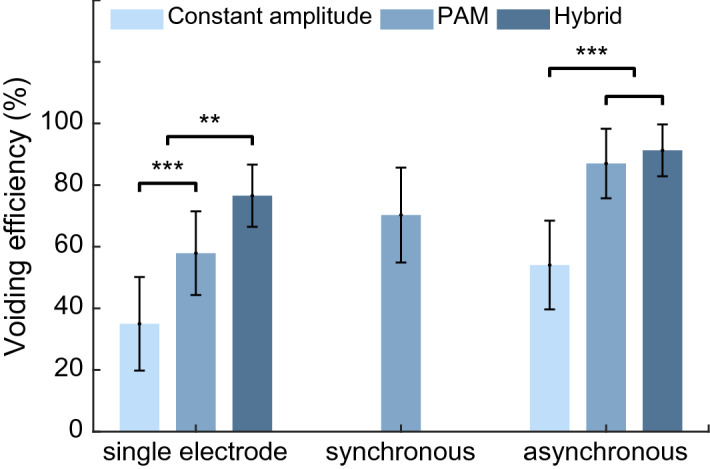
Summary of the voiding efficiency obtained using the closed-loop control of ISMS with single electrode stimulation and two-electrode synchronous and asynchronous stimulation. During the stimulation pattern with fixed amplitude, the pulse amplitude was kept constant at 80 μA. During closed-loop control with pulse amplitude modulation (PAM), the ISMS is controlled by the pulse amplitude of the stimulation. During hybrid closed-loop control, the ISMS is controlled by both pulse amplitude and pulse frequency. ($$**p<0.01, ***p<0.001$$, N-way ANOVA).

Figure 12 summarizes the voiding efficiency obtained using the closed-loop control of ISMS with different stimulation strategies. The results indicate that the single-electrode closed-loop control with hybrid stimulation strategy provides significantly higher voiding efficiency than that with PAM and constant strategies ($p<0.01,$ N-way ANOVA). With PAM strategy, there is no significant difference between two-electrode synchronous stimulation and single-electrode ($p=0.2579,$ two-way ANOVA), whereas the two-electrode asynchronous stimulation achieved significantly higher efficiency than the two-electrode synchronous ($p=0.0006,$ two-way ANOVA).

## Discussion

In this paper, we proposed a closed-loop control strategy for efficient bladder voiding using ISMS without requiring rhizotomy, neurotomy, or high-frequency blocking. Intermittent stimulation is alternately applied to the two electrodes that are implanted in the S2 lateral ventral horn and S1 dorsal gray commissure, to excite the bladder motoneurons and to inhibit the urethral sphincter motoneurons. It was suggested that the pontine micturition center coordinates the bladder voiding via a direct excitatory connection both with the bladder detrusor muscle motoneurons and with the the GABAergic inhibitory interneurons in the dorsal gray commissure that inhibits the EUS motoneurons^38^. Unlike the previous closed-loop controllers^32,33^, which are based on the stimulation of PN^32^ and Pelvic nerve^33^, the current study is based on the ISMS. One of the functional hallmarks of ISMS is the ability to simulate the neural networks within the spinal cord that could coordinate the contraction of the bladder and the EUS and avoid some of the difficulties associated with direct peripheral nerve stimulation^31^ (i.e., DSD). The ISMS may provide a promising approach for bladder contraction with EUS relaxation without requiring rhizotomy^7^, neurotomy^15,21,24^, or high-frequency blocking. Moreover, ISMS could preferentially recruit fatigue-resistant muscle fibers and generates gradual force^39–41^. The gradual force recruitment characteristics of ISMS have been attributed to its ability to activate motoneurons in a near normal physiological order based on their sizes.

An important issue in closed-loop control of bladder function is the feedback information. In the previous closed-loop control of bladder voiding^32,33^, the bladder pressure was used as the feedback information. Although, the bladder pressure is simple to record, it is affected by abdominal pressure changes, such as those initiated by coughs, laughs, and sneezes^42^. Moreover, simple changes in posture could have an effect on the vesical pressure.

To cope with the limitation of the implantable artificial bladder sensors, several methods have been proposed to estimate either the IVP or volume using electroneurography of the pudendal nerve^43–45^, electroneurography of the pelvic nerve^46^ and sacral nerve roots^46,47^, and neural activity recorded from the dorsal horn of the spinal cord^48,49^. In previous work^49^, we developed a method based on deep neural network for simultaneous estimating both pressure and volume from the neural activity recorded directly from the spinal cord gray matter neurons. Combining the method developed in this paper with our previous work^49^ via implanting a microelectrode array in the spinal cord for both recording and stimulation, could be considered for a future study.

An attractive feature of the current study is that the proposed control strategy does not require any threshold setting for triggering the stimulation or for classifying the bladder states (i.e. voiding and non-voiding), while in previous stuty^32^, two pressure thresholds were required to determine for triggering the stimulation. In another study^33^, a bladder state classification was first performed using a defined threshold of IVP. Then, the current amplitude was adjusted based on the estimated bladder state.

Fabrication and implantation of the intraspinal microwires are other challenges facing the ISMS. Additionally, implanting microwires into the body always carries the risk of infection and migration. However, recent progress in microwire fabrication and knowledge gained from implantable electrodes on animals^36,50–52^ can be applied to the development of a chronically implanted prosthetic device.

As a concluding remark, this work represents a promising approach to develop closed-loop neuroprosthetic systems for restoring bladder function in individuals with spinal cord injury or neurological disorders. The proposed closed-loop strategy is based on multielectrode asynchronous stimulation of ISMS. The results show that the multielectrode asynchronous paradigm provides effective bladder voiding at a lower level of bladder pressure and a lower level of stimulation amplitude compared to the single-electrode or two-electrode synchronous paradigms. Achieving effective voiding at a low level of pressure is clinically attractive because high bladder pressure may lead to upper tract deterioration. Moreover, the multielectrode asynchronous paradigm provides the possibility to activate asynchronously different groups of motoneurons within a motor pool and to reduce muscle fatigue.

## Data Availability

All data needed to evaluate the conclusions in the paper are present in the paper and/or the Supplementary Materials. All datasets generated during the current study are available from the corresponding author on reasonable request (erfanian@iust.ac.ir).

## References

[1] Abrams P (2003). The standardisation of terminology in lower urinary tract function: Report from the standardisation sub-committee of the International Continence Society. Urology.

[2] Gulur DM, Drake MJ (2010). Management of overactive bladder. Nat. Rev. Urol..

[3] Drake MJ, Williams J, Bijos DA (2014). Voiding dysfunction due to detrusor underactivity: An overview. Nat. Rev. Urol..

[4] Stoffel JT (2016). Detrusor sphincter dyssynergia: A review of physiology, diagnosis, and treatment strategies. Transl. Androl. Urol..

[5] Wyndaele J-J (2016). The management of neurogenic lower urinary tract dysfunction after spinal cord injury. Nat. Rev. Urol..

[6] Koprowski, C. *et al.* Urodynamic evaluation of adult neurogenic lower urinary tract dysfunction: a review. *Am. J. Urol. Res.* **2**, 29–37, Preprint at https://www.scireslit.com/Urology/download.php?file=AJUR-ID20.pdf (2017).

[7] Liao, L. & Madersbacher, H. *Neurourology: Theory and Practice*. (Springer Netherlands, 2019). 10.1007/978-94-017-7509-0

[8] Chapple, C. R., Wein, A. J. & Osman, N. I. *Underactive Bladder*. (Springer International Publishing, 2017). 10.1007/978-3-319-43087-4

[9] Bartley J, Gilleran J, Peters K (2013). Neuromodulation for overactive bladder. Nat. Rev. Urol..

[10] Lee JW (2015). Emerging neural stimulation technologies for bladder dysfunctions. Int. Neurourol. J..

[11] Coolen R, Groen J, Blok B (2019). Electrical stimulation in the treatment of bladder dysfunction: Technology update. Med. Devices Evid. Res..

[12] Brindley GS, Polkey CE, Rushton DN, Cardozo L (1986). Sacral anterior root stimulators for bladder control in paraplegia: The first 50 cases. J. Neurol. Neurosurg. Psychiatry.

[13] Yoo PB, Woock JP, Grill WM (2008). Bladder activation by selective stimulation of pudendal nerve afferents in the cat. Exp. Neurol..

[14] McGee MJ, Grill WM (2014). Selective co-stimulation of pudendal afferents enhances bladder activation and improves voiding efficiency. Neurourol. Urodyn..

[15] Peng C-W, Chen J-JJ, Cheng C-L, Grill WM (2008). Improved bladder emptying in urinary retention by electrical stimulation of pudendal afferents. J. Neural Eng..

[16] Woock JP, Yoo PB, Grill WM (2008). Activation and inhibition of the micturition reflex by penile afferents in the cat. Am. J. Physiol. Integr. Comp. Physiol..

[17] Tai C, Roppolo JR, de Groat WC (2004). Block of external urethral sphincter contraction by high frequency electrical stimulation of pudendal nerve. J. Urol..

[18] Tai C, Roppolo JR, de Groat WC (2005). Block of external urethral sphincter contraction by high frequency biphasic electrical stimulation of pudendal nerve. J. Urol..

[19] Bhadra N, Bhadra N, Kilgore K, Gustafson KJ (2006). High frequency electrical conduction block of the pudendal nerve. J. Neural Eng..

[20] Tai C, Wang J, Wang X, Roppolo JR, de Groat WC (2007). Voiding reflex in chronic spinal cord injured cats induced by stimulating and blocking pudendal nerves. Neurourol. Urodyn..

[21] Boger A, Bhadra N, Gustafson KJ (2008). Bladder voiding by combined high frequency electrical pudendal nerve block and sacral root stimulation. Neurourol. Urodyn..

[22] Boger AS, Bhadra N, Gustafson KJ (2012). High frequency sacral root nerve block allows bladder voiding. Neurourol. Urodyn..

[23] Peh WYX (2018). Novel neurostimulation of autonomic pelvic nerves overcomes bladder-sphincter dyssynergia. Front. Neurosci..

[24] Boggs JW, Wenzel BJ, Gustafson KJ, Grill WM (2006). Bladder emptying by intermittent electrical stimulation of the pudendal nerve. J. Neural Eng..

[25] Tai C, Wang J, Wang X, de Groat WC, Roppolo JR (2007). Bladder inhibition or voiding induced by pudendal nerve stimulation in chronic spinal cord injured cats. Neurourol. Urodyn..

[26] Bruns TM, Bhadra N, Gustafson KJ (2008). Variable patterned pudendal nerve stimuli improves reflex bladder activation. IEEE Trans. Neural Syst. Rehabil. Eng..

[27] Bruns TM, Bhadra N, Gustafson KJ (2009). Bursting stimulation of proximal urethral afferents improves bladder pressures and voiding. J. Neural Eng..

[28] McGee MJ, Grill WM (2016). Temporal pattern of stimulation modulates reflex bladder activation by pudendal nerve stimulation. Neurourol. Urodyn..

[29] Grill WM, Bhadra N, Wang B (1999). Bladder and urethral pressures evoked by microstimulation of the sacral spinal cord in cats. Brain Res..

[30] Tai C, Booth AM, de Groat WC, Roppolo JR (2004). Bladder and urethral sphincter responses evoked by microstimulation of S2 sacral spinal cord in spinal cord intact and chronic spinal cord injured cats. Exp. Neurol..

[31] Pikov V, Bullara L, McCreery DB (2007). Intraspinal stimulation for bladder voiding in cats before and after chronic spinal cord injury. J. Neural Eng..

[32] Lin Y-T (2014). Dual-channel neuromodulation of pudendal nerve with closed-loop control strategy to improve bladder functions. J. Med. Biol. Eng..

[33] Peh WYX (2018). Closed-loop stimulation of the pelvic nerve for optimal micturition. J. Neural Eng..

[34] Matsuura S, Downie JW (2000). Effect of anesthetics on reflex micturition in the chronic cannula-implanted rat. Neurourol. Urodyn..

[35] Harrison M (2013). Vertebral landmarks for the identification of spinal cord segments in the mouse. Neuroimage.

[36] McCreery D, Pikov V, Lossinsky A, Bullara L, Agnew W (2004). Arrays for chronic functional microstimulation of the lumbosacral spinal cord. IEEE Trans. Neural Syst. Rehabil. Eng..

[37] McGee, M. J. Effects and mechanisms of patterned electrical stimulation of pudendal afferents for bladder control. *ProQuest Dissertations and Theses* (Duke University, 2015), Preprint at https://dukespace.lib.duke.edu/dspace/bitstream/handle/10161/9818/McGee_duke_0066D_12748.pdf?sequence=1

[38] Blok BFM, de Weerd H, Holstege G (1997). The pontine micturition center projects to sacral cord GABA immunoreactive neurons in the cat. Neurosci. Lett..

[39] Bamford JA, Putman CT, Mushahwar VK (2011). Muscle plasticity in rat following spinal transection and chronic intraspinal microstimulation. IEEE Trans. Neural Syst. Rehabil. Eng..

[40] Snow S, Horch KW, Mushahwar VK (2006). Intraspinal microstimulation using cylindrical multielectrodes. IEEE Trans. Biomed. Eng..

[41] Bamford JA, Putman CT, Mushahwar VK (2005). Intraspinal microstimulation preferentially recruits fatigue-resistant muscle fibres and generates gradual force in rat. J. Physiol..

[42] Karam R (2015). Real-time classification of bladder events for effective diagnosis and treatment of urinary incontinence. IEEE Trans. Biomed. Eng..

[43] Wenzel BJ, Boggs JW, Gustafson KJ, Grill WM (2006). Closed loop electrical control of urinary continence. J. Urol..

[44] Wenzel, B. J., Grill, W. M., Boggs, J. W. & Gustafson, K. J. Detecting the onset of hyper-reflexive bladder contractions from pudendal nerve electrical activity. *Conf. Proc. Annu. Int. Conf. IEEE Eng. Med. Biol. Soc. IEEE Eng. Med. Biol. Soc. Annu. Conf.* **2004**, 4213–6. 10.1109/IEMBS.2004.1404175 (2005). 10.1109/IEMBS.2004.1404175 17271233

[45] Mathews KS (2014). Acute monitoring of genitourinary function using intrafascicular electrodes: Selective pudendal nerve activity corresponding to bladder filling, bladder fullness, and genital stimulation. Urology.

[46] Jezernik S, Wen JG, Rijkhoff NJM, Djurhuus JC, Sinkjaer T (2000). Analysis of bladder related nerve cuff electrode recording from preganglionic pelvic nerve and sacral roots in pigs. J. Urol..

[47] Kurstjens GAM, Borau A, Rodriguez A, Rijkhoff NJM, Sinkjaer T (2005). Intraoperative recording of electroneurographic signals from cuff electrodes on extradural sacral roots in spinal cord injured patients. J. Urol..

[48] Park JH (2013). Detecting bladder fullness through the ensemble activity patterns of the spinal cord unit population in a somatovisceral convergence environment. J. Neural Eng..

[49] Jabbari M, Erfanian A (2019). Estimation of bladder pressure and volume from the neural activity of lumbosacral dorsal horn using a long-short-term-memory-based deep neural network. Sci. Rep..

[50] Bamford JA, Marc Lebel R, Parseyan K, Mushahwar VK (2017). The fabrication, implantation, and stability of intraspinal microwire arrays in the spinal cord of cat and rat. IEEE Trans. Neural Syst. Rehabil. Eng..

[51] Toossi A, Everaert DG, Azar A, Dennison CR, Mushahwar VK (2017). Mechanically stable intraspinal microstimulation implants for human translation. Ann. Biomed. Eng..

[52] Toossi A (2018). Ultrasound-guided spinal stereotactic system for intraspinal implants. J. Neurosurg. Spine.

